# The Effects of Traditional Chinese Music and Western Classical Music on Mental Fatigue Induced by Cognitive Tasks

**DOI:** 10.3390/bs16020277

**Published:** 2026-02-14

**Authors:** Shuyue Tan, Ruxin Li, Leyi Zhang, Jialin Fan

**Affiliations:** 1 School of Psychology, Shenzhen University, Shenzhen 518060, China18998377895@163.com (R.L.);; 2 The Shenzhen Humanities & Social Sciences Key Research Bases of the Center for Mental Health, Shenzhen 518060, China

**Keywords:** music intervention, mental fatigue, cross-cultural comparison, cognitive performance

## Abstract

Mental fatigue refers to subjective feelings ranging from tiredness to exhaustion that appear after or during prolonged periods of cognitive activity. Music could be a powerful tool for relieving mental fatigue due to its beneficial effects on attention, which tend to decline when mental fatigue occurs. Moreover, traditional Chinese music is usually neglected and rarely used in music intervention studies, although its potential has been mentioned in China’s domestic journals. Therefore, the present study aimed to investigate the effects of traditional Chinese music and Western classical music on laboratory-induced mental fatigue. Three groups of Chinese non-psychology undergraduate students were assessed in terms of alertness, hedonic tone, and overall fatigue via a pre/post-intervention diary, a visual analogue mood scale, and the psychomotor vigilance test. The results showed that both traditional Chinese music and Western classical music mitigated fatigue-related declines in alertness and hedonic tone, and produced shorter reaction times, although there was no significant difference between the effects of the types of music.

## 1. Introduction

Music, an irreplaceable form of entertainment for many people, is more than just gratifying rhythms on certain occasions. In the modern world, where fatigue is a common occurrence, music can be a powerful tool for relieving driving fatigue, exercise-induced fatigue, and cancer-related fatigue (e.g., R. Li et al., 2019; Lal & Craig, 2000; J. Li & Wang, 2008; Qi et al., 2021). As societies have shifted from physical to mental labor over time, cases of mental fatigue have been on the rise (Boksem & Tops, 2008; Loge et al., 1998; Pawlikowska et al., 1994). However, until now, there has been little research into the effect of music interventions on mental fatigue.

Mental fatigue refers to subjective aversive feelings ranging from tiredness to exhaustion, which appear after or during prolonged periods of cognitive activity (Frone & Tidwell, 2015; Ream & Richardson, 1996). Mentally fatigued individuals usually express a decreased ability to focus and regulate emotions (Boksem et al., 2005; Hofmann et al., 2012). They may experience difficulties shifting attention away from unimportant information and subsequently spend more time on the task at hand (Boksem et al., 2005; Faber et al., 2012). The Limited Resource Model suggests that emotion regulation relies on the same energy pool as cognitive tasks (Baumeister et al., 1998). Therefore, for those whose mental resources have been depleted, it is challenging to suppress the negative emotions induced by external stimuli (Grillon et al., 2015) and consequently experience more intense negative emotions (D. Wang et al., 2022).

Drawing on attention-based models of mental fatigue and applying the framework of Attention Restoration Theory (ART) (Ohly et al., 2016), music interventions are hypothesized to support attentional regulation and recovery from mental fatigue impairment (Ding et al., 2025; Z. Wang et al., 2025). Research shows relaxing music can potently reduce fatigue-induced attentional impairment (Guo et al., 2015). Notably, classical music requires greater concentration than pop music and is typically engaged in as a solitary activity (Hanser et al., 2016). One possible explanation is that music elevates physical arousal, which is termed the “Mozart effect” (Mammarella et al., 2007; Thompson et al., 2001). Furthermore, listening to certain types of music, such as classical music, has been demonstrated to enhance spatial cognitive abilities and working memory capacity (Aheadi et al., 2010; Ivanov & Geake, 2003; Mammarella et al., 2007). These enhancements may make task completion less demanding overall, thereby conserving mental resources for attentional processes. Notably, these attentional and cognitive benefits vary by individual, as factors like music preference and familiarity modulate emotional and cognitive engagement (Dickson & Schubert, 2019).

Complementing the cognitive perspective, the efficacy of music in mitigating mental fatigue can also be predicted through its emotion-regulatory capacity. Given that mental fatigue is inherently linked to a deterioration in mood, music serves as a compensatory resource. Music listening serves as a versatile emotion regulation tactic, with genre preferences aligning to specific goals: upbeat pop enhances positive moods, while classical music facilitates introspection (van Goethem & Sloboda, 2011; Cook et al., 2017). Listeners are able to process emotions conveyed in music (Swaminathan & Schellenberg, 2015), triggering activation in brain regions related to emotional processing (i.e., amygdala and hippocampus) (Halko et al., 2015; Koelsch, 2018), restoring the emotional balance disrupted by cognitive exertion. Collectively, music could address the affective dimension of mental fatigue.

Among all types of music, Chinese traditional music, or Chinese folk music, is usually overlooked in international journals in terms of its value in music interventions (Guo et al., 2015), although meaningful findings have been reported in China’s domestic journals. Traditional Chinese music appeared to be the most effective in alleviating sadness, compared with rock music and music pieces by Banderi (Lu et al., 2012), which may be attributable to the similarities between traditional Chinese music and Western music. Traditional Chinese music can be categorized into folk songs, Quyi (melodious art), folk instrumental music, and Xiqu instruments (traditional Chinese opera instruments) (J. Wang, 2022). Its scale is pentatonic, utilizing five tones: Gōng (“宫”), Shāng (“商”), Jué (“角”), Zhǐ (“徵”), and Yǔ (“羽”). These tones correspond theoretically to the Western solfège syllables do, re, mi, sol, and la, or the pitch classes C, D, E, G, and A in the key of C major. In terms of melodic structure, the Gong and Zhǐ modes resemble Western major scales, whereas the Yu mode is comparable to Western minor scales (Yang & Zheng, 2021). Scholars have suggested that listening to pieces composed with either major scales or the Gong and Zhǐ scales can evoke positive emotions, whereas pieces composed with minor scales and the Yu scale can induce negative emotions (Bai et al., 2016; Yang & Zheng, 2021). These findings imply that traditional Chinese music may be as effective as, if not more effective than, Western compositions at reducing negative emotions elicited by mental fatigue.

In light of the context discussed above, the present study aimed to evaluate the effects of traditional Chinese music and Western classical music on mental fatigue, using a multidimensional assessment approach. We aimed to investigate whether these interventions could mitigate the decline in both subjective energy levels and objective cognitive vigilance. Specifically, we hypothesized that: (1) Exposure to music (either Traditional Chinese or Western Classical) during the break would lead to a significant reduction in mental fatigue compared with a no-music control condition. (2) This alleviation would be evidenced by improved self-reported ratings and preserved psychomotor performance. (3) We also sought to explore potential differences in efficacy between the two music genres. Additional measures, including mood states, were collected to provide a broader characterization of fatigue-related changes.

## 2. Methods

### 2.1. Participants

This study recruited non-music and non-psychology undergraduate students from Shenzhen University as participants. To ensure adequate statistical power, a priori power analysis was conducted using G*Power software 3.1. With an effect size of 0.3, an alpha level of 0.05, and power set at 0.80, a minimum sample size of 31 participants per group was required. To account for potential attrition, 63 students initially enrolled. To minimize the impact of music preference, participants who showed no preference for Chinese or Western classical music in the music preference questionnaire were invited to participate in the experiment (*n* = 48). All participants were right-handed with normal vision and auditory perception. The average age was 19.27 ± 1.47 years. Thirty out of 48 participants were female. Participants were randomly allocated to one of three groups: traditional Chinese music, Western classical music, or a silent control group (*n* = 16 per group). To ensure gender balance across groups, a stratified randomization procedure was implemented using a computer-generated allocation sequence prepared by an independent researcher. All of them participated voluntarily after being informed of the procedure of the study and provided signed informed consent before the beginning of the study. The School of Medicine Institutional Review Board at Shenzhen University reviewed and approved this study.

### 2.2. Materials

Mental fatigue was evaluated through both subjective and objective measures. Subjective fatigue and mental states were assessed using self-reported diary ratings and Visual Analogue Scales (VASs), while objective fatigue-related performance was assessed using reaction time measures from the Psychomotor Vigilance Test (PVT).

#### 2.2.1. Fatigue Induction: The 2-Back Task

**Figure 1:**
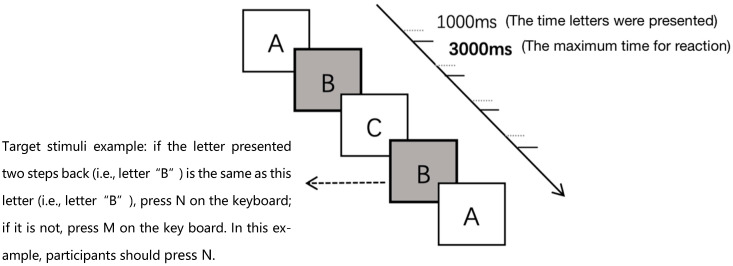
2-back task paradigm. Letters were presented sequentially in the center of the screen. Participants were required to indicate whether the current letter matched the one presented two steps back by pressing the corresponding key. Each stimulus was displayed for 1000 ms, with an inter-stimulus interval of up to 3000 ms.

N-back is a continuous performance task used to assess working memory, attention, and intelligence (Miller et al., 2009); the tool can also be applied as a mental fatigue-inducing tool (e.g., Ai et al., 2017; Ishii et al., 2013). The present study used the 2-back task and not the 1-back task, which is simplistic, or the 3-back task, which might cause the overconsumption of cognitive resources (Tanaka et al., 2009). Twenty-six letters in the modern English alphabet were selected as stimuli. During the task, letters in black were shown randomly in the center of the screen with a white background. The participants were asked to decide whether the letter currently appearing on the screen was the same as the penultimate number counting from it. If it was, they were asked to press *N* on the keyboard; if it was not, they were required to press *M* on the keyboard. Participants were asked to react as soon as possible. Each stimulus was presented for 1000 ms, and the maximum time for participants to react was 3000 ms (see Figure 1).

#### 2.2.2. Music Materials: Traditional Chinese Music and Western Classical Music

Generally, low-tempo music (under 90 beats per minute, BPM) holds unique stress-relieving properties by promoting physical relaxation and improving emotional well-being (Zhou et al., 2023; Fernández-Sotos et al., 2016). Specifically, it can improve moods while moderating physiological arousal, such as heart rates, during stressful situations (Yamamoto et al., 2007). Within this category of music, low-tempo classical music stands out as particularly soothing and effective for stress reduction (Imbir & Gołąb, 2016; Yamamoto et al., 2007). These mechanistically grounded insights not only elucidate music’s role in counteracting mental fatigue, but also informed our cross-cultural music selection strategy. Specifically, low-tempo Western classical and Chinese traditional compositions were prioritized based on these shared characteristics and empirically validated stress-reduction profiles.

For consistency, only instrumental pieces were chosen for the present study. The following music tracks were sourced from NetEase Cloud Music, China. For the traditional Chinese music group, Chinese instrumental ensembles 7 pieces included “Gao Shan Liu Shui” (“High Mountains and Flowing Waters”), “Cai Yun Zhui Yue” (“Colorful Clouds Chasing the Moon”), and “Chun Jiang Hua Yue Ye” (“A Moonlit Night on the Spring River”) performed and recorded by prominent Chinese orchestras or soloists. For the Western classical music group, according to the “Mozart effect”, we selected Mozart’s 5 pieces, including “Eine Kleine Nachtmusik, K. 525 II. Romance,” “Clarinet Concerto in A major, K. 622: II. Adagio” and “Concerto for flute and harp in C major, K.299.” A complete copy of the music list is provided in Supplementary Material S1.

The volume of all the excerpts was between 50 dB and 60 dB, with a tempo between 52 BPM (Adagio) and 84 BPM (Moderato). Music editing software was used selectively to adjust the volume and tempo of the music excerpts. All the selected pieces were played continuously for 40 min in a single, non-repeating sequence, the order predetermined by ascending original duration. The volume of all the music materials was measured by a BZ-7222 (Brüel & Kjær, Copenhagen, Denmark) sound level meter before the beginning of the experiment. All the participants wore MDR-ZX110AP headphones (SONY, Tokyo, Japan) to listen to the music.

#### 2.2.3. The Pre/Post-Intervention Diary

We used the Chinese version of the before work diary in the pre/post-work diary designed by A. P. Smith and Smith (2017) and translated and adjusted by Fan (Fan et al., 2021). The diary contains five items. The first three items are in the pre-diary section: sleep hours, pre-intervention fatigue, and sleep quality. The last two items are listed in the post-diary section: post-intervention fatigue and workload. Each item was rated on a scale of 1 to 10, with higher numbers indicating higher intensities of the participants’ feelings. The diary was printed on paper and given to the participants. A copy of the diary in its original language and in Chinese is provided in Supplementary Material S2.

#### 2.2.4. Visual Analogue Scales (VASs)

The VAS is an assessment tool for mental fatigue and is considered the most practical (M. R. Smith et al., 2019). The VAS adopted in the present study was used in previous studies (e.g., Allen & Smith, 2015). A total of 18 items are included in the VAS, each of which was a bipolar scale of zero to nine, with two opposing adjectives describing mood on the extremes. Numbers closer to the extremes represent feelings closer to the corresponding adjectives. The participants were asked to choose the values that best aligned with their current mood. The component scales for alertness and hedonic tone were selected for the measurement of mental fatigue. The items included in the alertness scale were “drowsy/alert, strong/feeble, coordinated/clumsy, attentive/dreamy, lethargic/energetic, muzzy/clear-headed, incompetent/proficient, and mentally slow/quick-witted.” The component scale for hedonic tone included “contented/discontented, happy/sad, antagonistic/friendly, interested/bored, self-centered/outward going, and withdrawn/sociable” (Allen & Smith, 2015, p. 2). A copy of the VAS in its original language and in Chinese is provided in Supplementary Material S3.

#### 2.2.5. Psychomotor Vigilance Test (PVT)

**Figure 2:**
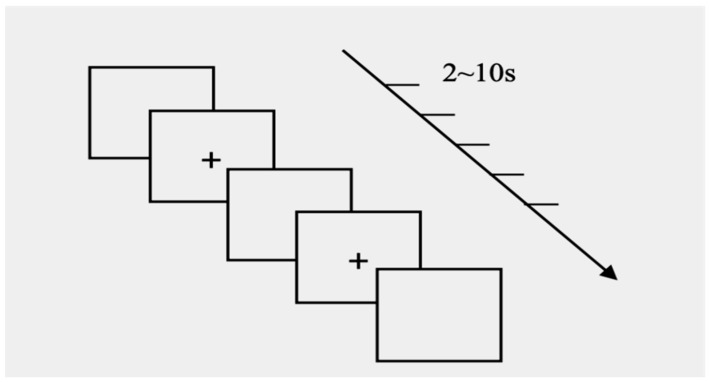
PVT paradigm used to assess behavioral alertness. A visual stimulus (“+”) appeared at random intervals (2–10 s). Participants were instructed to respond as quickly as possible by clicking the left mouse button upon its appearance.

The PVT is a mentally demanding test used to assess reaction time and vigilance. It has been used to assess cognitive performance and mental fatigue in many studies (Dimitrakopoulos et al., 2018; Fan et al., 2021; X. Wang et al., 2022). The present study adopted the PVT to assess the participants’ baseline and post-intervention task abilities. The duration of this test was set at 10 min because the standard length of the PVT lies between 10 and 20 min (Sun et al., 2014). The symbol “+” was shown randomly in the middle of the screen at intervals of 2–10 s. Participants were asked to click the left mouse button immediately after identifying the symbol. They were also informed that prediction was not allowed and that a sign on the screen, “False Starts” (FS), would appear if the prediction was detected. Participants completed the task in a quiet environment (see Figure 2).

### 2.3. Procedure

Considering possible individual differences in fatigue baselines at different times during a typical day, participants in each group were evenly arranged to participate in the study either in the morning or afternoon. The basic financial compensation was set at 70 CNY, with participants explicitly informed prior to the study that an additional 0–20 CNY would be awarded contingent upon their task compliance levels. Individuals who signed up for the experiment, met the inclusion criteria, and provided signed informed consent participated in the study.

**Figure 3:**
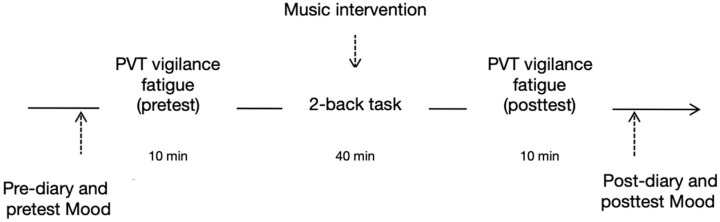
Flowchart of the experimental procedure. Participants were first assessed using a pre-diary, Visual Analogue Scales (VASs), and a PVT task. They then underwent a 40 min fatigue induction phase via the 2-back task, during which the experimental groups listened to assigned music (Traditional Chinese or Western Classical) while the control group performed the task in silence. This was immediately followed by a post-intervention PVT task and the completion of the VAS and post-diary.

At the beginning of the study, the participants were asked to complete the pre-diary, the VAS, and a 10 min PVT task. Then, in the fatigue induction stage, the 2-back task was conducted continuously for 40 min, during which the selected traditional Chinese and Western classical music excerpts were played through the headphones provided to all participants by the researchers. Meanwhile, participants in the control group completed the 2-back task in a quiet environment (no music). After the fatigue induction stage, the participants were asked to perform another 10 min PVT task before filling out the VAS and the post-diary (see Figure 3).

### 2.4. Data Analysis

The data were analyzed using SPSS 23.0. Descriptive statistics were computed for the dependent variables. The current experiment adopted a two-factor mixed design. The first independent variable was the between-group factor “music type,” which included three levels: “traditional Chinese music,” “Western classical music,” and “no music.” The second independent variable was the within-factor “time”, which included pre-test (before) and post-test (after). The dependent variables were “overall fatigue,” “alertness,” and “hedonic tone.” A one-way ANOVA was computed for the five questions in the diary questionnaire. A 2 × 3 (time: before, after; music type: traditional Chinese music, Western classical music, no music). ANOVA was conducted for the average reaction time of the PVT tasks and the self-rated alertness and hedonic tone. Effect sizes for repeated ANOVA model is reported as partial eta squared, which reflects the proportion of variance explained by a given factor after controlling for other effects in the model. Additionally, Bonferroni corrections were applied to post hoc and simple effects analyses involving multiple comparisons.

## 3. Results

Independent-sample *t*-tests revealed no significant differences among the three groups regarding age, gender, sleep duration, sleep quality, pre-intervention fatigue, baseline alertness, and baseline PVT reaction time (all *p* > 0.05), confirming that the random assignment was effective.

Nine dependent variables were identified, and descriptive statistics are presented in Table 1. A significant positive correlation was found between diary fatigue (post) and pre- and post-test PVT reaction times (*p* < 0.05), Alertness (pre-test) and Hedonic tone (pre-test) (*p* < 0.05), as well as pre-test PVT reaction times and post-test reaction times (*p* < 0.05), showed statistically significant positive associations.

### 3.1. The Effect of Music on Overall Fatigue

A one-way analysis of variance (one-way ANOVA) test was conducted for the overall fatigue changes obtained by subtracting the pre-diary scores from the post-diary scores. No significant difference was detected between the fatigue changes of any group (see Table 2). A repeated measures ANOVA test was conducted for the diary fatigue (pre) and diary fatigue (post) scores. The main effects of music types were non-significant (*p* > 0.05). The overall diary fatigue (post) score was significantly higher than diary fatigue (pre) score (*F* = 47.213, *p* < 0.01, *η*^2^ = 0.512).

To better explain whether there were significant differences between fatigue in different groups. Paired-sample *t*-tests conducted revealed a significant increase in subjective fatigue for the control group (t = −5.519, *p* < 0.05, MD = 3.37, 95% CI [2.072, 4.678]) and we also observed difference in the music group (t = −5.368, *p* < 0.05, MD = 2.72, 95% CI [1.686, 3.752]) (see Table 3). These results confirm the 2-back task’s effectiveness in inducing mental fatigue. Given the multiple paired-sample comparisons, a Bonferroni-adjusted significance threshold (α = 0.025) was applied to control for family-wise error. Paired-sample *t*-tests showed a significant increase in diary fatigue from pre- to post-task, which remained significant after Bonferroni correction (*p* < 0.01).

### 3.2. The Effect of Music on Alertness

The alertness of the participants was assessed via the scores of alertness, which were the sum of items in the alertness subscale of the VAS results, and PVT results served as an objective indicator of sustained attention and cognitive fatigue. The average score of alertness (after) (M = 26.08, SD = 7.12) was significantly lower than that of alertness (before) ((M = 39.31, SD = 4.22), *F*(1, 45) = 204.759, *p* < 0.05, *η*^2^ = 0.820).

A repeated measures ANOVA with the alertness scores of all the groups as the dependent variable was conducted (Table 4). The main effect of the music types was significant (*F*(2, 45) = 7.481, *p* < 0.01, *η*^2^ = 0.250), with no difference between the two music groups but significantly lower scores in the control group (*p* < 0.05). Significant interaction effects were detected between music types and time (*F*(2, 45) = 10.241, *p* < 0.01, *η*^2^ = 0.313). Simple effects analysis revealed that all experimental music conditions were associated with a significantly reduced decline in alertness from pre- to post-test, whereas the control group exhibited a larger decrease (*p* < 0.05).

In terms of objectively measured alertness, the control group solely contributed to the PVT average reaction time (after) (M = 385.24, SD = 6.60), which was significantly longer than the PVT average reaction time (before) (M = 340.31, SD = 40.14; *F*(1, 45) = 60.882, *p* < 0.01, *η*^2^ = 0.575) (see Table 4).

To better explain whether there were significant differences between average reaction time in different groups. Paired-sample *t*-tests conducted states revealed a significant increase in subjective reaction time for the control group (t = −10.222, *p* < 0.05, MD = 110.067, 95% CI [89.638, 130.495]) and we also observed difference in the music group (t = −2.012, *p* < 0.1, MD = 10.447, 95% CI [−5.078, 25.972]) (see Table 3). These results also effectively reflects the degree of mental fatigue in individuals.

A 2 (time: before, after) × 3 (music types: no music, Western classical music, traditional Chinese music) repeated measure ANOVA was conducted for the average reaction time of the PVT task of all the participants. The main effects of the music types were significant (*F*(2, 45) = 6.023, *p* < 0.01, *η*^2^ = 0.211). Significant interaction effects were found between the music types and time (*F*(2, 45) = 29.703, *p* < 0.01, *η*^2^ = 0.569). In addition, simple effects analysis showed that only the control group exhibited a significant difference in pre- and post-test average PVT reaction times (*p* < 0.05).

### 3.3. The Effect of Music on Hedonic Tone

The hedonic tone scores were the sum of all six items in the hedonic tone subscale of the VAS. A 2 (time: before, after) × 3 (music types: no music, Western classical music, traditional Chinese music) repeated measure ANOVA was conducted (Table 4). The average score of hedonic tone (before) (M = 39.71, SD = 4.68) was significantly higher than that of hedonic tone in the post-task section ((M = 33.79, SD = 6.67), *F*(1, 45) = 52.529, *p* < 0.01, *η*^2^ = 0.042). The overall hedonic tone of the traditional Chinese music group and the Western classical music group was higher than the control group (*p* < 0.05). A significant interaction effect was detected between the music types and time, *F*(2, 45) = 25.511, *p* < 0.01, *η*^2^ = 0.531. Simple effect analysis showed that only the control group scored significantly different on time (see Table 4).

## 4. Discussion

The current study found that both traditional Chinese music and Western classical music mitigated fatigue-related impairments in alertness and emotional affect compared to no music conditions. The effects of music intervention were examined at both the subjective and objective levels. The 2-back task successfully induced fatigue, as shown by increased self-reported fatigue and objective vigilance declines in PVT reaction times, aligning with established n-back paradigms (Ishii et al., 2013; M. R. Smith et al., 2019). The PVT’s sensitivity to fatigue-related impairment confirmed its utility as a robust behavioral measure, a critical methodological strength. Post-intervention analyses showed that both music groups displayed similar advantages over the control group in attentional performance (PVT reaction time) and hedonic tone, suggesting comparable effectiveness of Chinese and Western musical traditions in buffering fatigue-related effects.

Within the framework, subjective fatigue ratings reflect perceived attentional depletion, whereas task-based measures index sustained attentional demand over time. The presence of mental fatigue in all participants was not reflected in diary fatigue assessed before and after the intervention. The differences between the scores of diary fatigue (pre) and diary fatigue (post) were not significant between the control group and the two music groups. In other words, participants who received either traditional Chinese music interventions or Western music interventions reported feeling as fatigued as those who had not received any intervention. This presents an dissociation between subjective exhaustion and objective performance. While music may not immediately reverse the subjective perception of being “tired” (captured by the diary measure), it may buffer the functional consequences of mental fatigue.

Notably, the effectiveness of the 2-back task in inducing mental fatigue was reflected in participants’ alertness and hedonic tone. The control group reported feeling significantly less alert and happy and spent more time on the PVT after completing the 2-back task. Therefore, the failure of the diary measure to reflect group differences in mental fatigue may be attributable, at least in part, to the limited sensitivity of the single-item assessment. One self-rated question before and after a mental fatigue induction task might not represent the participants’ true feelings.

The beneficial role of music on mental fatigue was corroborated by three main findings. First, the participants in the traditional Chinese music group and the Western classical music group were more alert and attentive after the mental fatigue-inducing section than those in the control group. Second, the participants in the music groups spent less time reacting to the stimuli in the PVT than those in the control group. Third, only the participants in the control group had lower hedonic tones (i.e., reported feeling less happy) after the 2-back task. Taken together, these findings suggest that both traditional Chinese music and Western classical music may help counteract the adverse effects of mental fatigue on attention and emotional state. However, it is worth noting that the impact of these two types of music seems comparable. The participants in the traditional Chinese music group were as alert and pleasurable as those in the Western classical music group.

Several strengths exist in the present study. Foremost, by combining subjective self-reports (diary and VAS) with an objective behavioral task (PVT), we enhanced the robustness of our conclusions. We also provide empirical data pertaining to the effects of traditional Chinese music on mental fatigue, alertness, and hedonic tone, which are new in the field of music interventions. The therapeutic properties of Chinese music, as suggested in prior literature (Lu et al., 2012), may stem from its elastic “rubber-band rhythm” that mimics natural human speech patterns, combined with pentatonic scales (宫/gong, 商/shang, 角/jiao, 徵/zhi, 羽/yu) that create harmonious, non-dissonant soundscapes (Bai et al., 2016; Yang & Zheng, 2021). With this information, we also presented the feasibility and value of adopting traditional Chinese music in mental fatigue interventions. Further, the information regarding Western classical music in the present study supplements what has already been discovered (e.g., Guo et al., 2015; Jacquet et al., 2021). However, both musical traditions appear to be similarly effective in mitigating mental fatigue, suggesting that their beneficial effects may stem from shared features such as slow tempo, instrumental nature, and structural predictability, rather than from culturally specific compositional techniques. Nevertheless, this does not preclude the potential cultural significance of music in shaping user engagement, emotional resonance, and contextual acceptability, warranting profound investigations into the value of traditional Chinese music as well as music from other cultural contexts in future research.

The present study also suffered from several weaknesses. The variables included in the present study can be divided into two groups: self-rated variables and behavioral variables. Although such variables could be easily measured and quantified, the results were inevitably limited, as these variables relied heavily on the participants’ judgments and compliance. The involvement of electroencephalography recordings might be more convincing and powerful. While the study applied Bonferroni correction to control for family-wise error, the modest sample size may have limited statistical sensitivity to small effects, in addition to the inherent constraints of self-reported and behavioral measures. Moreover, we placed emphasis mainly on indicators closely related to mental fatigue (i.e., alertness and emotion). In other words, the direct measurement of subjective feelings of mental fatigue was neglected, as it was only assessed through the scores of one item in the diary questionnaire. In addition, the lack of diversity in the participants, who were all university students born and raised in China, might have contributed to potential biases in the results. It is highly possible that the Chinese participants have frequently been in contact with traditional Chinese music, which might lead to a higher familiarity of the participants with the selected traditional Chinese music materials and, subsequently, bias the results. Thus, future studies should sample participants from varied cultural backgrounds and systematically examine the role of musical preference, as favorite, uplifting tracks can markedly boost endorphin and dopamine release. Furthermore, the fact that the present study was conducted in a laboratory might have influenced the results to be less representative of real-life scenarios. Therefore, the effects of music interventions should also be examined in field settings, such as in a noisy classroom or a quiet library.

## 5. Conclusions

The present study assessed the effects of traditional Chinese music and Western classical music on mental fatigue induced by the 2-back task. The measurements involved subjective and objective assessments (i.e., diary, VAS, and PVT) of participants’ alertness, hedonic tone, and overall mental fatigue. The results showed that both traditional Chinese music and Western classical music mitigated mental fatigue impairments in attention and emotional state, although no significant difference was detected between the two types of music. The present study is innovative in terms of applying cross-cultural music materials to mental fatigue interventions. Furthermore, it provided a new perspective in studies of mental fatigue by corroborating the value and potential of traditional Chinese music as a material for fatigue interventions.

## Figures and Tables

**Table 1:** Descriptive statistics and correlation analysis of dependent variables.

	Mean	Standard Deviation	Diary Fatigue (Pre)	Diary Fatigue (Post)	Alertness (Before)	Alertness (After)	PVT Average Reaction Time (Before)	PVT Average Reaction Time (After)	Hedonic Tone (Before)	Hedonic Tone (After)
Diary Fatigue (pre)	3.792	1.957	1							
Diary Fatigue (post)	6.417	1.955	0.084	1						
Alertness (before)	39.313	4.223	−0.334 *	−0.387 **	1					
Alertness (after)	26.083	7.119	−0.107	−0.209	0.189	1				
PVT average reaction time (before)	340.308	40.139	0.019	0.321 *	−0.015	0.215	1			
PVT average reaction time (after)	385.240	62.601	0.088	0.363 *	0.025	−0.259	0.397 **	1		
Hedonic tone (before)	39.708	4.686	−0.069	−0.449 **	0.605 **	−0.016	−0.072	0.138	1	
Hedonic tone (after)	33.792	6.675	−0.010	−0.363 *	0.231	0.601 **	0.169	−0.435 **	0.018	1

* *p* < 0.05, ** *p* < 0.01.

**Table 2:** One-way ANOVA of diary variables.

	Music Types (Mean ± SD)			*F*	*p*	*η* ^2^
	Chinese Classical Music Group	Western Classical Music Group	Control Group			
Sleep Hours	7.34 ± 1.33	6.88 ± 0.90	7.38 ± 0.83	1.156	0.324	0.049
Sleep Quality	7.31 ± 1.54	7.75 ± 1.61	8.00 ± 1.46	0.819	0.447	0.035
Diary Fatigue (pre)	4.06 ± 1.88	3.63 ± 2.31	3.69 ± 1.74	0.226	0.798	0.010
Diary Fatigue (post)	6.19 ± 2.07	6.00 ± 2.22	7.06 ± 1.44	1.367	0.265	0.057
Diary Fatigue (post-pre)	2.13 ± 2.66	2.38 ± 3.20	3.38 ± 1.93	0.999	0.376	0.043
Workload	5.63 ± 2.53	5.75 ± 1.65	6.13 ± 1.82	0.519	0.599	0.023

**Table 3:** Paired-sample *t*-tests of diary fatigue and PVT average reaction time.

	*n*	M ± SD	t	Cohen’s d	Sig.
Control group					
Diary Fatigue (pre)	16	2.88 ± 1.41	−5.519	1.38	<0.001
Diary Fatigue (post)	16	6.25 ± 1.65			
PVT average reaction time (before)	16	336.274 ± 25.161	−10.222	2.56	<0.001
PVT average reaction time (after)	16	443.924 ± 51.946			
Music intervention group					
Diary Fatigue (pre)	32	4.25 ± 1.98	−5.368	0.95	<0.001
Diary Fatigue (post)	32	6.97 ± 1.86			
PVT average reaction time (before)	32	342.325 ± 46.084	−2.012	0.36	0.053
PVT average reaction time (after)	32	355.897 ± 44.380			

**Table 4:** Repeated measure ANOVA of music types and time, with alertness, PVT average reaction time, and hedonic tone as the dependent variable.

	Type III Sum of Squares	df	Mean Square	*F*	Sig.	Partial Eta Squared
Alertness						
Music types	468.271	2	234.135	7.481	0.002	0.250
Time	4200.260	1	4200.260	204.759	0.000	0.820
Music types * Time	420.146	2	210.073	10.241	0.000	0.313
PVT average reaction time						
Music types	37,339.858	2	18,669.929	6.023	0.005	0.211
Time	48,451.603	1	48,451.603	60.882	0.000	0.575
Music types * Time	47,276.939	2	23,638.470	29.703	0.000	0.569
Hedonic tone						
Music types	567.250	2	283.625	12.479	0.000	0.357
Time	840.167	1	840.167	52.529	0.000	0.539
Music types * Time	816.083	2	408.042	25.511	0.000	0.531

## Data Availability

The data supporting this study are not publicly available due to privacy and confidentiality restrictions, as they contain sensitive information that could compromise the anonymity and privacy of the research participants. The data are, however, available from the corresponding author upon reasonable request and subject to approval by the relevant ethics committee.

## Supplementary Materials

The following supporting information can be downloaded at: https://www.mdpi.com/article/10.3390/bs16020277/s1, S1: The list of musical pieces selected, S2: The diary in English and Chinese, S3: VAMS in English and Chinese.
