# Supplementary material for: The Effects of Traditional Chinese Music and Western Classical Music on Mental Fatigue Induced by Cognitive Tasks
Source: Behav Sci (Basel). 2026 Feb 14;16(2):277. doi: 10.3390/bs16020277 (PMC12938388; doi:10.3390/bs16020277)
Supplement: Supplementary file 1 [file behavsci-16-00277-s001.zip › behavsci-4047273-supplementary.pdf]

Supplementary Material S1

The list of musical pieces selected

Category	Musical pieces	Composer	Performer	Track duration
**Traditional Chinese music**	Gao Shan Liu Shui	/	CHINA RECORD CORPORATION	07:59
	Cai Yun Zhui Yue	/	China Broadcasting Chinese Orchestra	03:30
	Chun Jiang Hua Yue Ye	/	Central Chinese Orchestra	09:19
	Yu Zhou Chuan Wan	/	Chinese National Orchestra	04:48
	Mei Hua San Nong	/	China National Traditional Orchestra	05:07
	Zui Yu Chang Wan	/	Chinese National Orchestra	04:28
	Han Gong Qiu Yue	/	Dehai Liu	07:00
**Western classical music**	Eine Kleine Nachtmusik, K. 525 II. Romanc	Mozart	New Philharmonia Orchestra	06:08
	Clarinet Concerto in A major, K. 622: II. Adagio	Mozart	Royal Philharmonic Orchestra	08:52
	Concerto for flute and harp in C major, K.299	Mozart	Berliner Philharmoniker	11:20
	Piano Concerto No. 21, K. 467: II. Andante	Mozart	The Cleveland Orchestra	07:47
	Piano Concerto No. 23 in A Major, K. 488:II. Adagio	Mozart	Maria Joao Pires / Gulbenkian Foundation Symphony Orchestra Lisbon / Theodore Guschlbauer	07:20

Note：”/” indicates that no single composer can be identified; the piece is of traditional origin.

Supplementary Material S2

A) The diary in its original language:

1. How many hours sleep did you get last night?

This question asks about your recent sleep experience, no matter it was at daytime or at night.

________ hours ________ minutes

2. How was the quality of your sleep?

Not at all good Very good

1 2 3 4 5 6 7 8 9 10

3. How long did it take you to travel to work?

________ hours ________ minutes

4. How fatigued did you feel from your commute?

Not at all Very fatigue

1 2 3 4 5 6 7 8 9 10

5. How well are you feeling now?

Not at all well Very well

1 2 3 4 5 6 7 8 9 10

6. How alert do you feel now?

Not at all Very alert

1 2 3 4 5 6 7 8 9 10

B) The diary in Chinese:

1. 您昨晚睡了几个小时？ _________小时

2. 您昨晚的睡眠质量如何？

一点都不好

非常好

1 2 3 4 5 6 7 8 9 1 0

3. 您现在感觉有多疲劳？

一点都不疲劳

非常疲劳

1 2 3 4 5 6 7 8 9 1 0

4. 您现在整体感觉如何？

一点都不好

非常好

1 2 3 4 5 6 7 8 9 1 0

5. 您现在自我感觉有多清醒/机敏/警觉？

非常困倦/迷糊 非常

清醒/警觉

1 2 3 4 5 6 7 8 9 1 0

Note: As the study participants were non-employed college students whose daily routines did not involve workplace commuting, Question 3 of the questionnaire (How long did it take you to travel to work?) was removed to ensure ecological validity between the measurement instrument and the target population.

Supplementary Material S3

A) VAMS in its original language:

Alerteness:

Drowsy	0	1	2	3	4	5	6	7	8	9	Alert
Relaxed	0	1	2	3	4	5	6	7	8	9	Excited
Strong	0	1	2	3	4	5	6	7	8	9	Feeble
Muzzy	0	1	2	3	4	5	6	7	8	9	Clear-headed
Coordinate	0	1	2	3	4	5	6	7	8	9	Clumsy
Lethargic	0	1	2	3	4	5	6	7	8	9	Energetic

Hedonic tone:

Contented	0	1	2	3	4	5	6	7	8	9	Discontented
Troubled	0	1	2	3	4	5	6	7	8	9	Tranquil
Mentally slow	0	1	2	3	4	5	6	7	8	9	Quick-witted
Tense	0	1	2	3	4	5	6	7	8	9	Calm
Attentive	0	1	2	3	4	5	6	7	8	9	Dreamy
Incompetent	0	1	2	3	4	5	6	7	8	9	Proficient
Happy	0	1	2	3	4	5	6	7	8	9	Sad
Antagonistic	0	1	2	3	4	5	6	7	8	9	Friendly
Interested	0	1	2	3	4	5	6	7	8	9	Bored
Withdrawn	0	1	2	3	4	5	6	7	8	9	Sociable
Depressed	0	1	2	3	4	5	6	7	8	9	Elated
Self-centered	0	1	2	3	4	5	6	7	8	9	Outward going

B) VAMS in Chinese:

Alerteness:

困倦的	0	1	2	3	4	5	6	7	8	9	亢奋的
放松的	0	1	2	3	4	5	6	7	8	9	兴奋的
强壮的	0	1	2	3	4	5	6	7	8	9	虚弱的
迷糊的	0	1	2	3	4	5	6	7	8	9	清醒的
手脚协调的	0	1	2	3	4	5	6	7	8	9	笨手笨脚的
昏昏欲睡的	0	1	2	3	4	5	6	7	8	9	精力充沛的

Hedonic tone:

满足的	0	1	2	3	4	5	6	7	8	9	不满的
困扰的	0	1	2	3	4	5	6	7	8	9	平静的
思维迟钝的	0	1	2	3	4	5	6	7	8	9	反应灵活的
紧张的	0	1	2	3	4	5	6	7	8	9	冷静的
专心的	0	1	2	3	4	5	6	7	8	9	恍惚的
能力不足的	0	1	2	3	4	5	6	7	8	9	专业的
快乐的	0	1	2	3	4	5	6	7	8	9	忧伤的
敌对的	0	1	2	3	4	5	6	7	8	9	友好的
充满兴趣的	0	1	2	3	4	5	6	7	8	9	感到无聊的
社交恐惧的	0	1	2	3	4	5	6	7	8	9	善于交际的
郁闷的	0	1	2	3	4	5	6	7	8	9	兴高采烈的
内向的	0	1	2	3	4	5	6	7	8	9	外向的
